# Assessing Age-Friendly Community Initiatives: Developing a Novel Survey Tool for Assessment and Evaluation

**DOI:** 10.1093/geroni/igaf122.293

**Published:** 2025-12-31

**Authors:** John Puxty

**Affiliations:** Queen’s University, Kingston, Ontario, Canada

## Abstract

**Background and Objectives:**

Age-friendly community initiatives (AFCIs) have gained recognition as essential responses to the needs of aging populations. Despite their growing significance, there is a notable lack of effective measurement tools to assess the planning, implementation, and sustainability of AFCIs. The purpose of this study was to develop and validate a survey tool for evaluating AFCIs.

**Research Design and Methods:**

A sequential exploratory mixed-method design was used in 2 phases. First, we identified key themes from interviews with AFCI leads to generate AFCI survey items and regional workshops. Then, we conducted a pilot of the survey and assessed its measurement properties.

**Results:**

Thematic analysis of interviews with 68 key informants from 58 AFCIs revealed 4 main themes: AFCI priorities, enablers, challenges, and benefits. These themes, combined with feedback from AFCI stakeholders at the regional workshops and an AFCI conference, informed the development and refinement of a reliable and valid AFCI survey in 2019, supported by a high Cronbach’s alpha value (α _= 0.881). Steps were identified to maintain and sustain the AFCI survey over time including during and post COVID Pandemic.

**Discussion and Implications:**

The survey accommodates AFCIs’ diverse demographics, governance structures, and priorities with a standardized and flexible approach for effective measurement. This research contributes to the academic understanding of AFCIs and aids community leaders and policymakers in planning, implementing, and evaluating AFCIs.

